# Retraction: A transcriptional signature associated with non-Hodgkin lymphoma in the blood of patients with Q fever

**DOI:** 10.1371/journal.pone.0347091

**Published:** 2026-04-13

**Authors:** The PLOS One Editors

The *PLOS One* Editors retract this article [1,2] due to concerns about compliance with the PLOS Human Subjects Research policy.

The Materials and Methods section in [1] reports that the study involved collection of blood samples from French patients with acute Q fever, *C. burnetii* persistent infection, *C. burnetii* lymphadenitis or *C. burnetii* with lymphoma as well as healthy donors (Établissement Français du sang). The article states that these tests were performed with the approbation of the ethical committee of the Aix-Marseille University, but it does not report an ethics approval reference number and it does not clarify the period during which the samples were collected.

The authors did not respond to the journal’s request for ethics approval documentation, and a representative of the Aix-Marseille Université Ethics Committee responded to the journal in June 2024 stating that the Scientific Integrity Unit of the University of Aix-Marseille was investigating the matter, but as of March 2026 PLOS has not received documentation confirming that prospective ethics approval was obtained for this study.

In the absence of the requested documentation, PLOS is unable to confirm the article’s compliance with the PLOS Human Subjects Research policy.

All authors either did not respond directly or could not be reached.
